# *In ovo* inhibition of avian pox virus replication by mangosteen rind and red ginger ethanolic extracts

**DOI:** 10.14202/vetworld.2021.2640-2645

**Published:** 2021-10-11

**Authors:** Annisaa’ Lu’lu Nur’aini, Sri Hartati, Tri Untari

**Affiliations:** 1 Postgraduate Program of Veterinary Science, Faculty of Veterinary Medicine, Gadjah Mada University, Yogyakarta, Indonesia; 2 Department of Internal Medicine, Faculty of Veterinary Medicine, Gadjah Mada University, Yogyakarta, Indonesia; 3 Department of Microbiology, Faculty of Veterinary Medicine, Gadjah Mada University, Yogyakarta, Indonesia.

**Keywords:** avian pox virus, *in ovo*, mangosteen rind, pock lesions, red ginger

## Abstract

**Background and Aim:**

Avian pox is a contagious disease caused by the avian pox virus (APV). Mangostin and γ-mangostin in mangosteen rind (MR) and gingerol in red ginger (RG) exhibit antiviral activity. In this study, we evaluated the effect of MR and RG ethanolic extracts on APV based on pock lesions on the chorioallantoic membrane (CAM) of specific pathogen-free (SPF) embryonated chicken eggs (ECEs).

**Materials and Methods:**

Three APVs from chicken isolates (C1, C2, and C3), one APV from a pigeon isolate (P), 1.5% and 3% MR ethanolic extract, 5% and 10% RG ethanolic extract, and a combination of 1.5% MR and 5% RG at 0.1 mL/egg were inoculated *in ovo* (7^th^ day incubation, chorioallantoic route) in SPF ECEs. A control group inoculated *in ovo* with APV alone was also established. Each treatment consisted of three replicates. Parameters including embryo survival, CAM lesions, and average number of pock lesions were determined.

**Results:**

*In ovo* inoculation of MR and RG ethanolic extracts was not harmful to the ECEs and did not induce CAM lesions. The average number of pock lesions in the control group (C1, C2, C3, and P) was 35, 14, 10, and 17, respectively, whereas in all treatment groups, the number was 0, except in the 5% RG group of C1, which had a value of 10.

**Conclusion:**

*In ovo* inoculation of 1.5% and 3% MR, 5% and 10% RG, and the combination of 1.5% MR plus 5% RG ethanolic extracts at 0.1 mL/egg inhibit APV by reducing the number of pock lesions on the CAM of the ECE.

## Introduction

Avian pox is an acute contagious disease caused by the avian pox virus (APV) [1,2]. This disease causes significant economic loss to domestic poultry because of decreased egg production, reduced growth, blindness, and increased mortality [3,4]. Mortality is usually low in birds with a mild cutaneous form of the disease; however, it becomes a significant problem with systemic infection when diphtheric lesions are dominant or when the disease is accompanied by other infections or poor environmental conditions [5-7]. Therapy for avian pox is not available; however, an alternative to control avian pox may lie in the use of medicinal plants [8,9].

Mangosteen rind (MR) (*Garcinia mangostana*) has been used as a traditional medicine for more than 100 years [10,11]. The main bioactive secondary metabolites of mangosteen are xanthone derivatives [12,13]. The primary constituents of the xanthone fraction include α-mangostin and γ-mangostin [14,15]. MR contains xanthones (mangostin and g-mangostin) which are non-competitive inhibitors of human immune deficiency virus-1 (HIV-1) protease by inhibiting the virus replication cycle [16]. Abimata [17] reported that MR extract (2%) can inhibit the replication of fowl pox virus *in ov*o. Ginger (*Zingiber officinale*) has been used as traditional medicine [18,19]. Ginger contains gingerol, which has been reported to inhibit HIV-1 replication at various stages of the viral life cycle in infected human MT4 T lymphocytes [20]. Ginger has also been shown to be effective against rhinoviruses [21], hepatitis C virus [22], herpes simplex virus type 1 (HSV-1) [23], and HSV type 2 [24]. Previously, the antiviral activity of a sea grape extract against APV was reported by Puspitasari[25]. However, no antiviral activity of red ginger (RG) or the combination of MR and RG against the APV has been reported.

Therefore, we evaluated the effect of MR and RG ethanolic extracts on APV based on pock lesions on the chorioallantoic membrane (CAM) of specific pathogen-free (SPF) embryonated chicken eggs (ECEs). Our findings will provide insight into new therapies for APV that can be used safely in chickens.

## Materials and Methods

### Ethical approval

No ethical committee approval was necessary for this study as we conducted the experiment on SPF ECEs. However, we conducted the experiment under very fine confinement without giving any undue stress to the birds.

### Study period and location

The study was conducted from August 2020 to December 2020. Mangosteen rind and red ginger rhizome for this study were obtained from Yogyakarta and processed at Laboratory of Pharmacy, Gadjah Mada University. Chickens and pigeon for this study were obtained from Yogyakarta. Isolation and identification of APV isolates and *in ovo* test were conducted at Laboratory of Virology, Center of Veterinary Wates. Histopathology examination was conducted at Laboratory of Pathology, Faculty of Veterinary Medicine, Gadjah Mada University. Polymerase Chain Reaction was conducted at Laboratory of Parasitology, Faculty of Veterinary Medicine, Gadjah Mada University.

### Isolation and identification of local APV isolates

Virus isolates were obtained from field cases in Yogyakarta (Indonesia). Three layer chickens and one pigeon with clinical symptoms of avian pox, as evidenced by nodular lesions on the comb, eye, wattle, and fibronecrotic lesions and on the mucosal membrane of the mouth of the pigeon, were collected [5]. The remaining samples of nodular lesions were stored in tightly closed conical tubes and labeled as C1 (Chicken 1), C2 (Chicken 2), C3 (Chicken 3), and P (Pigeon). Samples of nodular lesions for virus isolation were stored at 4°C. Samples for histopathological analysis were stored with 10% formaldehyde at room temperature (20-25°C) for 24 h [9].

Nodular and fibronecrotic lesions collected from chickens and pigeons displaying clinical symptoms of avian pox disease were ground separately with a sterilized mortar and pestle and then suspended in sterile phosphate-buffered saline to prepare a 10% suspension. The suspension was centrifuged at 7000 rpm for 10 min and the virus suspension was stored at −20°C [9]. Ten-day-old SPF ECEs were candled. The location of the air cavity and virus inoculation site was marked with a pencil, sterilized, and stabbed with a puncher. The air cavity was sucked in until a cavity was formed at the site of inoculation for the virus suspension. The holes in the air cavity and the location of virus inoculation were covered with tape and then the ECEs were incubated at 37°C for 24 h before virus inoculation on the CAM of the ECEs [26]. After 24 h, 4.5 mL of virus suspension was mixed with 0.5 mL of gentamicin (5.5 mg/mL viral suspension) and homogenized with a vortex for 30 s and incubated in a biosafety cabinet for 60 min [27]. Then, 0.2 mL of each viral suspension (C1, C2, C3, and P) was injected into the CAM of the ECEs. The puncture hole was closed with silicone gel [26]. Inoculated embryonic chicken eggs were placed on an egg rack and incubated at 37°C. Each dead ECE and ECEs that survived for up to 7 days after inoculation were placed into the refrigerator at 4°C for 24 h. CAM passage 1 was harvested and examined for pock lesions. The pock lesions on CAM were partially inserted into a tissue pot that containing 10% formalin for histopathology. The same method was followed until the fourth passage of virus propagation in CAM [9,26].

Samples for histopathological examination presented as nodular skin lesions in the pigeon (P) with clinical symptoms of avian pox and pock lesions on the CAM, which were harvested in the third (C1 and C3) and fourth (C2) passage in chickens. Histopathological preparations were done with the following stages: Fixation, trimming, pre-inclusion, embedding, sectioning, staining and mounting, and storage of paraffin blocks and slides, as described by Slaoui *et al*. [28].

Samples for polymerase chain reaction (PCR) included pock lesions on the CAM which were harvested at the first (P), third (C1 and C3), and fourth (C2) passage. Primers were designed based on the 4b gene sequence of the HP44 fowl pox virus (FPV) strain. Gen 4b is a core protein that functions as a structural protein [29,30]. The sequences of the primer sets were as follows: Forward, 5’-CAGCAGGTGCTAAACAACAA-3’ (identical to nucleotide 459-478), and reverse, 5’-CGGTAGCTTAACGCCGAATA-3’ (complementary to nucleotides 1016-1035). The size of the amplified DNA fragment was expected to be 578 bp [29]. DNA extraction was done with the PureLink™ Genomic DNA Mini Kit according to the manufacturer’s instructions. PCR was run according to a pre-determined program as follows: Denaturation (initial denaturation 94°C for 5 min, denaturation 94°C for 1 min), annealing (54°C for 30 s), and extension (extension 72°C for 30 s, final extension at 72°C for 5 min). Approximately 10–20 μL of the reaction were analyzed on a 1% agarosegel. The gel was observed for the presence of DNA using a gel documentation unit [31].

### MR and RG ethanolic extracts

MR and RG rhizome obtained from Yogyakarta (Indonesia) were extracted by a maceration procedure described by Indrawati *et al*. [32], which was adapted and scaled. Two kilograms of each fresh simplicia (RG rhizome and MR) were washed thoroughly in running water, sorted, and weighed. Fresh simplicia was oven-dried at 50°C for 48 h. The dry simplicia was ground in a grinding machine into a crude powder. The crude powder was extracted using the maceration method to obtain a 100% stock of RG and MR ethanolic extracts. RG powder (200 g) was transferred to a bottle, mixed with 1400 mL of 96% ethanol, and left for 24 h with stirring every hour. The extract was poured, the pulp was squeezed out with gauze, and separated into another bottle. The extract was evaporated in a water bath at 60°C with a fan to obtain a 9.7 g thick extract. The extract was cooled at 20-25°C. The same extraction method was used for the crude powder of MR. Each 100% stock of RG and MR ethanolic extract was poured into a conical tube and two dilutions were prepared by adding 1% carboxymethyl cellulose solution and sterile distilled water at 1:9 ratio. The solution was filtered through a Minisart^®^ Syringe Filter (0.45 μm) to obtain 1.5% and 3% MR ethanolic extracts, 5% and 10% RG ethanolic extracts, and an extract of 1.5% MR and 5% RG free of bacterial contamination [33].

### *In ovo* test

In an earlier study, Rasool *et al*. [33] showed that *in ovo* inoculation with a concentrated aqueous ginger extract (10%) showed antiviral activity against H_9_N_2_. Abimata [17] showed that MR extract (2%) inhibited the replication of fowl pox virus *in ovo*. In the present study, we used 1.5% and 3% MR ethanolic extract, 5% and 10% RG ethanolic extract, and a combination of 1.5% MR and 5% RG along with three APV of chicken isolates (C1, C2, and C3) and one APV of pigeon isolate (P), and SPF ECEs. The SPF ECEs were purchased from the Center of Veterinary Wates. Each treatment of APV infection (C1, C2, C3, and P) used three ECEs in Groups I-VII. MR and RG ethanolic extracts and APV were mixed in Eppendorf tubes according to group, vortexed for 30 s, and incubated for 60 min before inoculation into the CAM of ECEs. Group I was inoculated with 0.1 mL of 1.5% MR and 0.1 mL APV. Group II was inoculated with 0.1 mL of 3% MR and 0.1 mL APV. Group III was inoculated with 0.1 mL of 5% RG and 0.1 mL of APV. Group IV was inoculated with 0.1 mL of 10% RG and 0.1 mL of APV. Group V was inoculated with 0.05 mL of 1.5% MR mixed with 0.05 mL of 5% RG and 0.1 mL APV. Group VI was a positive control and was inoculated with 0.1 mL of APV. Group VII was a negative control and was not inoculated with the extract and APV. The treatments for Groups 1-VII were repeated for all samples. In addition, 15 ECEs were used in Group VIII as an herbal control for the herbal toxicity test and were inoculated with all extracts administered to the treatment group. The virus and extract inoculation method at this stage were the same as that for the virus isolation on the CAM of the ECEs. Inoculated embryonic chicken eggs were placed into an egg rack and incubated at 37°C. Each dead ECE and ECEs that survived for up to 7 days after inoculation were stored at 4°C for 24 h. The CAM was harvested and examined for pock lesions. Data were obtained from the macroscopic image of CAM and the number of pock lesions was counted on the CAM of the ECEs in all groups [9,26]. Group VIII, a control for the herbal and herbal toxicity test, was used to determine the maximum non-toxic concentration of each extract concentration [8,9].

### Statistical analysis

The results of *in ovo* tests (macroscopic images of CAM in Groups I-VIII, the number of CAM pock lesions in Groups I-VI, and the number of CAM lesions and the percentage of live embryos in Group VIII) were presented descriptively.

## Results

### APV isolation and identification

Nodular lesions on the comb, wattle, face, and body of chickens, nodular lesions on the eyelids and beak of pigeons, and fibronecrotic lesions on the mucous membranes of the pigeon’s mouth were used for *in ovo* inoculation of SPF ECEs. Morphological changes from individual pock lesions to thickening of the CAM were observed within 7 days after inoculation in three samples (C1, C2, and C3), whereas typical pox lesions were observed in pigeon isolates (P) during the first passage. The homogenate of the infected CAM of C1, C2, and C3 was used for CAM passage. After the third passage, typical pox lesions were observed in two samples (C1 and C3), whereas C2 required the fourth passage until the typical pox lesions were evident. In chicken isolate samples, white opaque pock formation was observed after the third passage. For pigeon isolate samples, the white opaque pock formation was already evident in the first passage.

The percentage of positive samples for avian pox according to species is shown in Table-1. Histopathology examination of the nodular skin lesions of the pigeon and CAM pock lesions of chickens revealed that all samples were positive for APV infection. The microscopic appearance of the skin of APV-infected pigeons exhibited proliferation of stratum spinosum cells and CAM infected with APV showed epithelial hyperplasia. The microscopic appearance of APV-infected CAM and skin also displayed intracytoplasmic body inclusions with ballooning degeneration in all samples. The PCR assay of the positive control (commercial APV vaccine “Medivac Pox Medion”) and all samples (C1, C2, C3, and P) were positive for APV, and no bands were observed in the gel for the negative control (ddH_2_0).

**Table-1:** Percentage of positive samples for avian pox according to species.

Animals	Samples	Test	Number	Positives (%)
Chickens	CAM pock lesions	Histopathology	3	100
		PCR	3	100
Pigeon	Nodular lesions	Histopathology	1	100
	CAM pock lesions	PCR	1	100

CAM=Chorioallantoic membrane, PCR=Polymerase chain reaction

### *In ovo* test

The toxic effects of various dilutions of MR and RG ethanolic extracts for chicken embryos are shown in Table-2. The toxicity of MR and RG ethanolic extracts was evaluated by an *in ovo* test. The percentage of live embryos treated with 1.5% and 3% MR ethanolic extract, 5% and 10% RG ethanolic extract, and a combination of 1.5% MR and 5% RG ethanolic extracts was 100%. Thus, the extracts were not toxic to ECEs and did not induce lesions on CAM on the 5^th^ day after inoculation.

**Table-2:** The toxic effects of different dilutions of mangosteen rind and red ginger ethanolic extracts for chicken embryos.

Ethanol extract	Live embryos (%)	CAM lesions
Mangosteen rind 1.5%	100	None
Mangosteen rind 3%	100	None
Red ginger 5%	100	None
Red ginger 10%	100	None
Combination of mangosteen rind 1.5% and red ginger 5%	100	None

CAM=Chorioallantoic membrane

**Figure-1:**
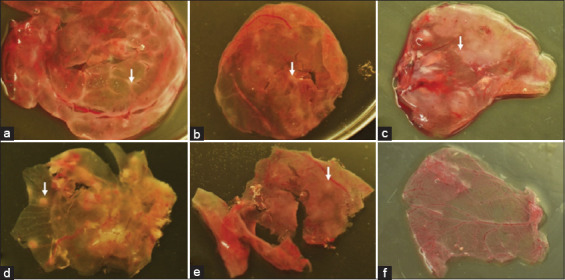
Effect of mangosteen rind and red ginger ethanolic extracts on avian post virus (APV) growth on chorioallantoic membrane of 10-day-old chicken embryos. The effects of different concentrations used are indicated (arrows) at; (a: chicken 1, b: chicken 2, c: chicken 3, d: pigeon) Positive control: Well-formed pocks; (e) Red ginger 5% with APV of chicken 1 isolate: Considerably reduced number of pocks; (f) Red ginger 10% with APV of chicken 1 isolate: No pocks were seen.

The antiviral activity of different dilutions of MR and RG ethanolic extracts against APV is shown in Table-3. The average number of pock lesions in the positive control group of the APV isolates (C1, C2, C3, and P) was 35, 14, 10, and 17, respectively. The average number of pock lesions in Groups I-V was 0 except for Group III, in which the average number of pock lesions for the 5% RG ethanolic extract and C1 was 10. Figure-1 shows well-formed pocks in the positive control group, whereas there were considerably reduced numbers of pocks of CAM in Group III (5% RG ethanolic extract and C1). The pock lesion was round, thickened, white, 3–5 mm in diameter, focal, and the CAM was thickened. No pock lesions were observed in Group IV (10% RG ethanolic extract and C1).

**Table-3:** Antiviral activity of different dilutions of mangosteen rind and red ginger ethanolic extracts against APV.

APV Isolates	The average number of pock lesions according to APV isolates					
	Positive control	Mangosteen rind		Red ginger		Combination of mangosteen rind 1.5% and red ginger 5%

		1.5%	3%	5%	10%	
Chicken 1	35	0	0	10	0	0
Chicken 2	14	0	0	0	0	0
Chicken 3	10	0	0	0	0	0
Pigeon	17	0	0	0	0	0

APV=Avian pox virus

## Discussion

Avian pox is frequently reported in free-range chickens in Yogyakarta (Indonesia). Although avian pox is well known to the indigenous farmers, the isolation and identification of avian pox has not been reported. We isolated this virus from clinical specimens of chickens and pigeon and showed the formation of pock lesions on the CAM of SPF ECEs. The presence of APV was confirmed by histopathology and PCR analysis. The pathogenicity of APV was retained when the APV of chickens and pigeon isolates was passaged on CAM and then inoculated into 10-day-old SPF ECEs. The viruses were successfully adapted to the CAM of the ECEs, which presented with typical pocks after one passage for APV in pigeon isolates and three to four passages for APV in chickens isolates. The APV of the pigeon isolate grew faster on the CAM of the ECEs. This may have occurred because pigeons had fibronecrotic (diphtheric) lesions on the mucosal membrane of the mouth, whereas samples from chickens 1, 2, and 3 only had nodular (cutaneous) skin lesions on the face and body. Mortality is usually low in birds with a mild cutaneous form of APV; however, mortality is high with systemic infection when diphtheric lesions are dominant [5-7]. Among the samples collected, 100% of the birds were positive for APV based on histopathology and PCR analysis.

The local APV isolates from the three chickens and one pigeon were used for an *in ovo* test. The maximal non-toxic concentration tolerated by chicken embryos was 3% for MR ethanolic extract and 10% for the RG ethanolic extract. MR and RG ethanolic extracts tested at various non-toxic concentrations exhibited strong antiviral activity against local APV isolates. Infected CAM from the positive control group displayed very clear pocks. A strong reduction in pock numbers was observed for all MR and RG ethanolic extract concentrations, which reduced the average number of pock lesions in chicken 1, 2, and 3 and pigeon isolates from 35, 14, 10, and 17, respectively, to 0. The 5% RG ethanolic extract of the chicken 1 isolate was only reduced from 35 to 10. This may have occurred because the symptoms in chicken 1 included both nodular lesions around the chicken’s face and on the body, whereas samples C2, C3, and P did not exhibit nodular lesions on the body. Thus, the severity of avian pox in chicken 1 was higher compared with the others.

The mechanism through which MR ethanolic extract reduces the number of APV pock lesion requires further study; however, research on the antiviral effects of MR ethanolic extract has been described previously. MR (*G. mangostana*) ethanolic extract contains xanthones (mangostin and γ-mangostin) which are non-competitive inhibitors of HIV-1 protease and inhibits the HIV-1 virus replication cycle [16]. Proteases also referred to as peptidases or proteinases, are enzymes involved in protein digestion. These abundant enzymes catalyze various proteolytic events that serve as mediators of signal initiation, transmission, and termination of cellular events, such as the inflammatory response, apoptosis, blood coagulation, and hormone processing. HIV protease inhibitors target mature proteases and disrupt the evolution of viral particles by stopping inhibiting the conversion of Gag and Gag-Pol by mature proteases. Therefore, viral development can be halted with the use of protease inhibitors [34]. Abimata [17] analyzed anti-FPV activity using an ethanolic extract of MR *in ovo*. The extract at a concentration of 2% was effective against FPV. The results of this study are consistent with that of Abimata [17]. We found that MR ethanolic extracts at concentrations of 1.5% and 3% exhibited strong antiviral effects, as evidenced by the reduced number of pock lesions in all APV samples.

The mechanism of RG ethanolic extract in reducing the number of APV pock lesions on the CAM of ECE also requires further study; however, the antiviral effects of ginger have been reported. Ginger rhizome (*Z. officinale*) contains gingerol, which inhibits the replication of HIV-1 during various stages of the viral life cycle in infected human MT4 T lymphocytes. Ingenol and gingerol assist CD4+ T cells in maintaining high cell viability to fight HIV-1 infection without disrupting viral replication [20]. Rasool *et al*. [33] showed that an aqueous ginger extract at a concentration of 10% was effective as an antiviral against AIV H_9_N_2_, whereas at a concentration of 5%, it had no antiviral effects. In contrast, we found that the RG ethanolic extract at a concentration of 5% had an antiviral effect against APV as evidenced by the reduced number of pock lesions, although they did not quite reach 0. These results are consistent with those of Rasool *et al*. [33] and Abimata [17] in which RG ethanolic extract at a concentration of 10% or the combination of MR 1.5% and RG 5% exhibited strong antiviral effects through a reduction in the number of pock lesions to 0.

## Conclusion

*In ovo* inoculation of 1.5% and 3% MR, 5% and 10% RG, or a combination of 1.5% MR and 5% RG ethanolic extracts at 0.1 mL/egg inhibits APV by reducing the number of pock lesions on the CAM of ECEs.

## Authors’ Contributions

ALN: Collected the samples, conducted the laboratory examinations, collected data, performed data analysis, and wrote the manuscript. SH and TU: Contributed to conceptualization, supervised the research, and reviewed and edited the manuscript. All authors read and approved the final manuscript.
